# Atomic insights into the material properties of double-perovskite-type hydride LiNaMg_2_H_6_ for H_2_ storage applications

**DOI:** 10.1039/d5ra05174f

**Published:** 2025-10-16

**Authors:** Son-Il Jo, Hyong-Ju Kim, Chol-Ho Pang, Un-Gi Jong, Tal-Hwan Kye

**Affiliations:** a Faculty of Materials Science and Technology, Kim Chaek University of Technology P.O. Box 76 Pyongyang Democratic People’s Republic of Korea jsi85922@star-co.net.kp; b Faculty of Materials Science, Kim Il Sung University P.O. Box 76 Pyongyang Democratic People’s Republic of Korea ug.jong@ryongnamsan.edu.kp

## Abstract

Perovskite-type compounds exhibit multi-functional properties that make them suitable for luminescence, photocatalysis, photovoltaics and H_2_ storage applications. Here, we provide atomic insights into the material properties of the double-perovskite-type hydride LiNaMg_2_H_6_ for H_2_ storage applications. Electronic structure calculations show that the cubic LiNaMg_2_H_6_ is an insulator with a direct band gap of 2.8 eV at the Γ point, consist with electron localization function and Born effective charge analyses. Based on geometric factors, elastic constants and self-consistent phonon calculations, we find that LiNaMg_2_H_6_ is dynamically and mechanically stable in the cubic phase at elevated temperatures, satisfying Born’s stability criteria. Finally, it is illustrated that the gravimetric and volumetric H_2_ storage capacities are 7.09 wt% and 91.12 g L^−1^, and the H_2_ desorption temperature is 548.54 K by considering the quantum effect, explaining well previous experimental observations. Our calculations highlight that LiNaMg_2_H_6_ hydride can be a potential H_2_ storage material because of its high H_2_ storage capacity, mechanical and dynamical stabilities and suitable H_2_ desorption temperature.

## Abstract 2

We systematically investigated the hydrogen storage properties of the double-perovskite-type hydride LiNaMg_2_H_6_, demonstrating the material’s stability, high storage capacities of 7.09 wt%, 91.12 g L^−1^, suitable dehydrogenation temperature of 540 K.

## Graphical abstract

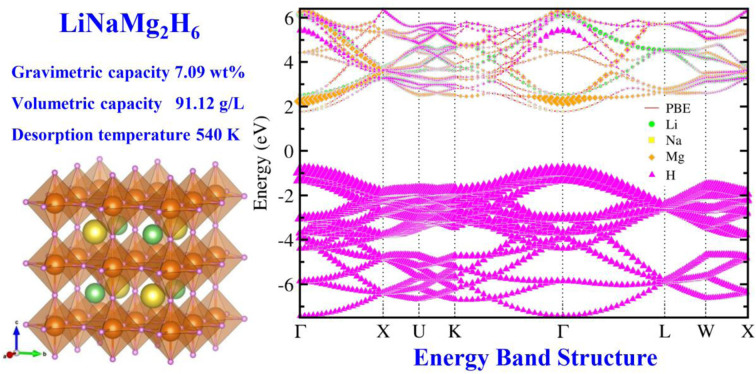

## 1 Introduction

The development of effective, cheap and safe hydrogen storage systems is one of the key areas for efficient operation of the hydrogen-based economy.^1,2^ This area has been intensively developing through new engineering solutions,^3,4^ whereas rapid technological progress is guaranteed by developing novel materials that can far outperform the currently used ones.

Perovskite-type compounds^5–8^ occupy a unique position in the design and discovery of new materials with targeted functionalities due to the wide range of constituent elements, diversity of crystal structures and distinctive physicochemical properties. In 1984, the double-perovskite-type compound Mg_2_FeH_6_ was first synthesized^9^ and reported to be a suitable candidate for thermochemical energy storage because of its high enthalpy change during hydride formation.^10^ Moreover, Zhang *et al.*^11^ illustrated that Mg and Fe nanoparticles absorb hydrogen in two steps, first forming MgH_2_ and then reacting with Fe and H_2_ to form Mg_2_FeH_6_, and it can release more than 5 wt% H_2_ in 10 min, indicating that Mg_2_FeH_6_ is a promising H_2_ storage material for H_2_-fueled vehicle applications. On the other hand, Ikeda *et al.* synthesized the single-perovskite-type hydride NaMgH_3_ by ball milling of NaH and MgH_2_ hydrides at ambient temperature and demonstrated the formation capability of other perovskite-type hydrides from a detailed analysis of geometrical factors.^12–15^ Importantly, they observed reversible H_2_ absorption and desorption process with a H_2_ gravimetric capacity of ∼6 wt% and fast H_2_ migration at elevated temperatures. Komiya *et al.*^16^ fabricated hydride perovskites of the form AMgH_3_ (A = Na, K, Rb) by mechanical milling, finding that the hydrides could release H_2_ at temperatures from 670 to 720 K *via* several different pathways depending on the specific A-site cation. Furthermore, Kou *et al.*^17,18^ revealed that the Zr and Co-based hydride perovskite ZrCoH_3_ can reversibly absorb and desorb H_2_, but, unfortunately, it suffers from the H_2_-induced disproportionation (HID) phenomena. However, they proved that the HID obstacle can be easily removed by partially substituting Zr or Co cations with Ti cations.

In addition to experimental investigations, many researchers have performed extensive theoretical studies^19–27^ in order to provide an in-depth understanding of the material properties of perovskite-type materials for H_2_ storage applications. Fornari *et al.*^26^ performed density functional theory (DFT) calculations to explore the structural and lattice dynamics properties of perovskite hydrides AMgH_3_ (A = Na, K, Rb), finding that the compounds exhibit an ionic bonding nature and dynamical stability. Based on *ab initio* calculations, Gencer *et al.*^8^ studied the electronic and mechanical properties of hydride perovskites ANiH_3_ (A = Li, Na, K) with H_2_ storage capacities of 4.4, 3.6 and 3.3 wt%, revealing their mechanical stability and metallic bonding nature. In addition, Siddique *et al.*^5,7^ confirmed the dynamical and chemical stabilities of hydride perovskites LiBH_3_ (B = Sc, Ti, V) and AVH_3_ (A = Be, Mg, Ca, Sr) with H_2_ storage capacities >4.0 wt% through DFT simulations. In 2024, Xu *et al.*^24^ reported that XAlH_3_ (X = Na, K) hydride perovskites show mechanical, dynamical and thermodynamical stabilities with metal-like electronic properties due to ionic chemical bonding.

Although there exist extensive investigations on perovskite-type compounds, little attention has been paid to the double-perovskite-type material LiNaMg_2_H_6_ for application as a H_2_ storage material. To the best of our knowledge following a review of the literature, only a few papers^28–30^ have studied the structure, thermal analysis and dehydriding kinetic properties of the Na_1−*x*_Li_*x*_MgH_3_ hydride, concluding that Li_0.5_Na_0.5_MgH_3_ (ref. 30) has better dehydriding kinetic properties and a higher H_2_ desorption amount of 4.11 wt% in comparison with NaMgH_3_. However, theoretical insights into the material properties were not fully uncovered for the double-perovskite-type hydride LiNaMg_2_H_6_ (*i.e.*, Li_0.5_Na_0.5_MgH_3_). The LiNaMg_2_H_6_ hydride has a high H_2_ gravimetric storage capacity over 7 wt% and, moreover, involves nontoxic and earth-abundant elements, and is therefore regarded as a potential candidate for high-performance, low-cost and clean H_2_ storage materials. Here, we provide atomistic insights into the material properties, including structural, electronic and lattice dynamics properties and dynamical and mechanical stabilities, of the double-perovskite-type hydride LiNaMg_2_H_6_ for H_2_ storage applications by using density functional theory calculations.

## 2 Computational methods

First-principles calculations were carried out employing the Vienna *ab initio* simulation package (VASP).^34,35^ In order to describe the interactions between ions and valence electrons, we used the projector augmented wave (PAW) potentials,^36,37^ where the valence electron configurations were given as Li-2s^1^, Na-3s^1^, Mg-3s^2^ and H-1s^1^. Based on a convergence test, it was revealed that a cutoff energy of 800 eV for the plane-wave basis sets and a *k*-point mesh of 10 × 10 × 10 provided a total energy accuracy of 2 meV per atom (see Fig. S1, SI). The variable-cell structural relaxations were performed until all the atomic forces were less than 10^−2^ eV Å^−1^ with a self-consistent convergence threshold of 10^−8^ eV. For the choice of exchange–correlation functional, we tested the Perdew–Burke–Ernerhof (PBE)^38^ and PBE-revised functionals for solids (PBEsol)^39^ within the generalized gradient approximation (GGA) and the Perdew–Wang (PW91)^40^ functional within the local density approximation (LDA) in order to account for the coulombic interactions among the valence electrons. We computed atomic forces for 2 × 2 × 2 supercells, using a reduced cutoff energy of 400 eV and a *k*-point mesh of 4 × 4 × 4 with the same convergence thresholds.

As suggested by Zhang *et al.*,^11^ the H_2_ desorption reaction for LiNaMg_2_H_6_ was considered as follows:

1 LiNaMg_2_H_6_ → Li + Na + 2Mg + 3H_2_.

Following eqn (1), we calculated the H_2_ desorption enthalpy Δ*H* as follows:

2 Δ*H* = *H*_Li_ + *H*_Na_ + 2*H*_Mg_ + 3*H*_H_2__ − *H*_LiNaMg_2_H_6__,

where *H*_compound_ is the enthalpy of the corresponding compound. Then, the enthalpy *H* can be calculated by considering the quantum effect as follows:

3 *H* = *E*_e_ + *E*_*z*_,

where *E*_e_ and *E*_*z*_ are, respectively, the DFT total energy and the zero-point energy considering the quantum effect. *E*_*z*_ was computed using the following formula:4
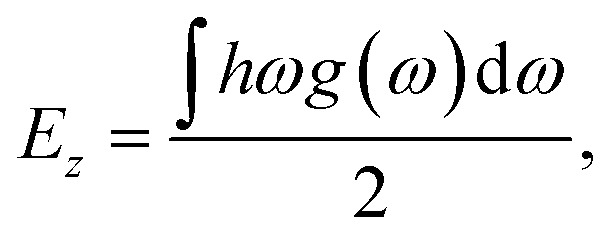
where *h*, *ω* and *g*(*ω*) are the Plank’s quantum constant, phonon frequency and phonon density of states, respectively. For the desorption of the total amount of hydrogen, the H_2_ desorption temperature *T*_d_ can be estimated as follows:

5 *T*_d_ = −Δ*H*_H_2__/Δ*S*,

where Δ*H*_H_2__ is the H_2_ desorption enthalpy per H_2_ molecule, which is calculated by dividing the desorption enthalpy Δ*H* (eqn (2)) by the number of H_2_ molecules *n* (*i.e.*, *n*= 3 for LiNaMg_2_H_6_), and Δ*S* denotes the change in entropy associated with the H_2_ desorption reaction described in eqn (1). In this work, the change in entropy Δ*S* was approximated as the entropy of H_2_ gas, *i.e.*, 130.7 J mol^−1^ K^−1^.^41^

The stability of H_2_ storage materials is one of the most important factors as it can play a crucial role in determining the durability and safety of H_2_ storage systems, such as H_2_-powered vehicles, fuel cells and energy storage mediums. Thus, we estimated the materials’ mechanical stability by evaluating elastic constants such as the bulk (*B*), shear (*G*) and Young’s (*E*) moduli. These elastic constants represent a material’s linear response to small strain. We computed the elastic stiffness (*C*_*ij*_) and compliance (*S*_*ij*_) constants based on density functional perturbation theory (DFPT)^42^ calculations. Materials in the cubic phase have three independent stiffness (compliance) constants, *C*_11_, *C*_12_ and *C*_44_ (*S*_11_, *S*_12_ and *S*_44_), from which the elastic moduli were estimated as follows:6
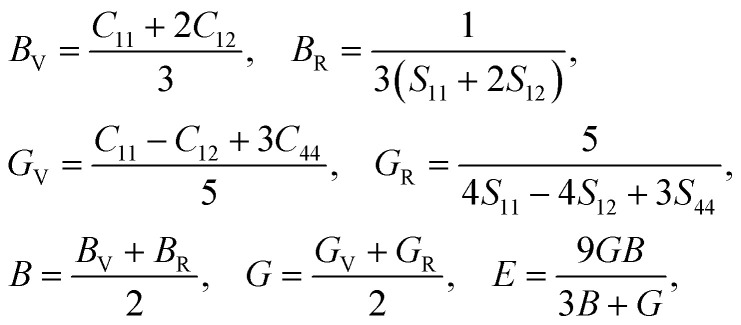
where the V and R subscripts represent the stiffness (compliance) constants within the Voigt and Reuss approximations, respectively. Polycrystalline solids in a cubic phase are mechanically stable when satisfying the Born’s stability criteria^43^ as follows:

7 *C*_11_ > 0, *C*_44_ > 0, *C*_11_ + 2*C*_12_ > 0, *C*_11_ − *C*_12_ > 0.

In addition, the elastic anisotropy is an important factor to assess the nature of microcrack sources and propagation in polycrystalline solids.^44^ The universal anisotropic index *A*^*U*^, the compression and shear percent anisotropies *A*_C_ and *A*_S_, and the shear anisotropic factor *A*_1_ are defined as follows:8
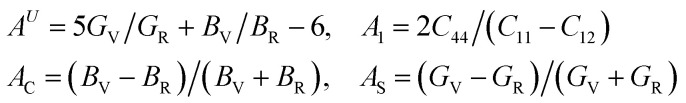


In the next step, we computed lattice dynamics properties including phonon dispersion curves and phonon density of states (DOS) to estimate *E*_*z*_ and the compound’s dynamical stability. The phonon dispersions and DOS calculations were carried out within the finite displacement method, as implemented in the ALAMODE code.^45,46^ Using 2 × 2 × 2 supercells (80 atoms), we generated 20 different configurations where all atoms were randomly displaced by 0.01 ∼ 0.06 Å from their equilibrium positions for LiNaMg_2_H_6_, with Li and Na in the cubic phase and Mg in the hexagonal phase. Then, the atomic forces were computed for all the displaced supercells by performing precise self-consistent calculations. The 2^nd^-, 3^rd^- and 4^th^-order force constants (IFCs) were calculated within the compressive sensing lattice dynamics (CSLD) approach^47^ using the ALAMODE code. We ensured that for all the compounds, the IFCs could reproduce the atomic forces with relative errors of less than 1.6% compared with the DFT-calculated forces (see Fig. S2, SI). We computed the harmonic (2^nd^-order) IFCs by considering all possible harmonic terms, from which the harmonic phonon dispersions and DOS were calculated at 0 K. Meanwhile, the 3^rd^- and 4^th^-order anharmonic IFCs were extracted by considering the 6^th^- and 3^rd^-nearest-neighbor interactions for each type of atom. By taking into account anharmonic effects at elevated temperatures, we calculated temperature-dependent phonon dispersions and DOS from the 3^rd^- and 4^th^-order IFCs within the self-consistent phonon (SCP) theory.^48^ Within the SCP theory, the anharmonic phonon eigenvalues were computed as functions of temperature from the pole of the Green’s function beyond the perturbation theory, as implemented in the ALAMODE code. In the SCP equation, the phonon self-energy becomes frequency-dependent only when the loop diagram associated with the quartic IFCs is considered. Note that the off-diagonal elements of the self-energy must be included to account for changes in phonon eigenvectors induced by anharmonic effects.

## 3 Results and discussion

### 3.1 Crystal structure properties

**Fig. 1:**
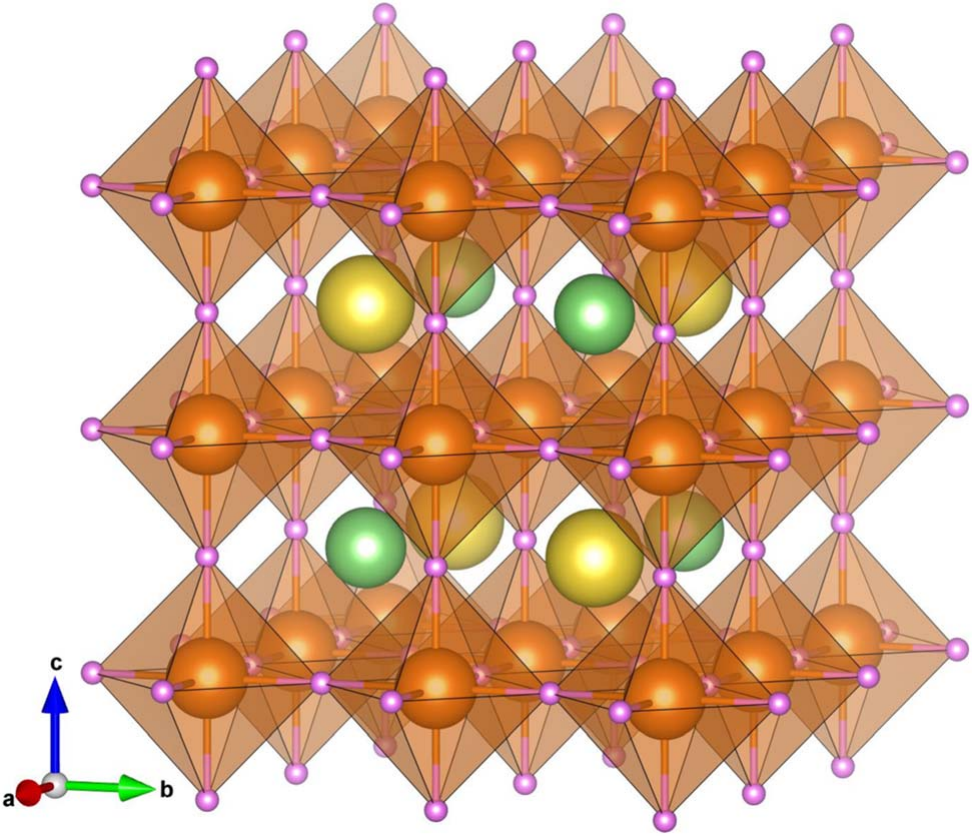
Polyhedral view of the crystalline structure optimized using the PBEsol functional for the double-perovskite-type hydride LiNaMg_2_H_6_ in the cubic phase with a space group of *Fm*3̄*m*. The green-, yellow-, brown- and purple-colored balls represent the Li, Na, Mg and H atoms, respectively.

Double-perovskite compounds have been found to successively crystallize in cubic, tetragonal and orthorhombic phases upon decreasing temperature. For instance, it was experimentally observed that double-perovskite-type hydrides A_2_TH_6_ (A = Ca, Sr, Ba; T = Fe, Ru, Os) adopt the cubic and lower-symmetry phases at different temperatures.^11,49^ Like in the single-perovskite-type hydrides ABH_3_, the stability of the double-perovskite-type hydride LiNaMg_2_H_6_ can be empirically assessed by using the Goldschmidt tolerance factor 
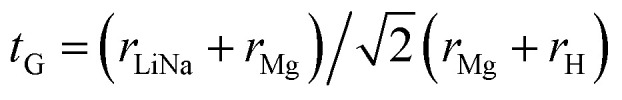
 and octahedral factor *t*_o_ = *r*_Mg_/*r*_H_, where *r*_LiNa_ is an average value of the ionic radii of Li^+^ and Na^+^, and *r*_Mg_ and *r*_H_ are the ionic radii of Mg^2+^ and H^−^ ions, respectively. It is generally accepted that *t*_G_ can be used to assess whether the A-site cation can fit between the BH_6_ octahedra, while *t*_o_ is used to check whether the BH_6_ octahedron is stable. According to the empirical criteria,^50,51^ compounds can adopt a stable perovskite-type structure when satisfying the criteria 1.0 ≥ *t*_G_ ≥ 0.7 and *t*_o_ ≥ 0.4.^52^ As listed in Table 1, the LiNaMg_2_H_6_ compound has suitable geometric factors of *t*_G_ = 0.82 and *t*_o_ = 0.65, being similar to the previous work.^53^ Based on the analysis of the geometric factors, we found that LiNaMg_2_H_6_ can stabilize in a perovskite-type structure. Therefore, we proposed that the LiNaMg_2_H_6_ hydride could adopt the cubic double-perovskite structure with the *Fm*3̄*m* space group like the oxide, halide and fluoride double-perovskite compounds (see Fig. 1). It should be noted that the geometrical factors such as *t*_G_ and *t*_o_ can provide a qualitative estimation of the phase stability, but a quantitative and detailed assessment of material stability requires precise calculations on the lattice dynamics and elastic properties.

**Table 1:** Lattice constants and atomic positions calculated using the PW91, PBE and PBEsol functionals and geometric factors *t*_G_ and *t*_o_ for LiNaMg_2_H_6_, Li, Na and Mg compounds with previous experimental data^31–33^

Compound	PW91					PBE				PBEsol				Exp.				Geometric factor	
	Lattice constants (Å)	Atom	Position			Lattice constants (Å)	Position			Lattice constants (Å)	Position			Lattice constants (Å)	Position				
			*x*	*y*	*z*		*x*	*y*	*z*		*x*	*y*	*z*		*x*	*y*	*z*	*t*_G_	*t*_o_
LiNaMg_2_H_6_	*a* = 7.58	Li	0.75	0.75	0.75	*a* = 7.62	0.75	0.75	0.75	*a* = 7.60	0.75	0.75	0.75	—	—	—	—	0.82	0.65
		Na	0.25	0.25	0.25		0.25	0.25	0.25		0.25	0.25	0.25	—	—	—	—		
		Mg_1_	0.50	0.50	0.50		0.50	0.50	0.50		0.50	0.50	0.50	—	—	—	—		
		Mg_2_	0.00	0.00	0.00		0.00	0.00	0.00		0.00	0.00	0.00	—	—	—	—		
		H_1_	0.75	0.25	0.25		0.75	0.25	0.25		0.75	0.25	0.25	—	—	—	—		
		H_2_	0.75	0.75	0.25		0.75	0.75	0.25		0.75	0.75	0.25	—	—	—	—		
		H_3_	0.75	0.25	0.75		0.75	0.25	0.75		0.75	0.25	0.75	—	—	—	—		
		H_4_	0.25	0.75	0.75		0.25	0.75	0.75		0.25	0.75	0.75	—	—	—	—		
		H_5_	0.25	0.75	0.25		0.25	0.75	0.25		0.25	0.75	0.25	—	—	—	—		
		H_6_	0.25	0.25	0.75		0.25	0.25	0.75		0.25	0.25	0.75	—	—	—	—		
Li	*a* = 3.44	Li_1_	0.50	0.50	0.50	*a* = 3.49	0.50	0.50	0.50	*a* = 3.47	0.50	0.50	0.50	*a* = 3.47^a^	0.50	0.50	0.50		
		Li_2_	0.00	0.00	0.00		0.00	0.00	0.00		0.00	0.00	0.00		0.00	0.00	0.00		
Na	*a* = 4.17	Na_1_	0.50	0.50	0.50	*a* = 4.25	0.50	0.50	0.50	*a* = 4.23	0.50	0.50	0.50	*a* = 4.22^b^	0.50	0.50	0.50		
		Na_2_	0.00	0.00	0.00		0.00	0.00	0.00		0.00	0.00	0.00		0.00	0.00	0.00		
Mg	*a* = 3.16	Mg_1_	0.66	0.33	0.75	*a* = 3.26	0.66	0.33	0.75	*a* = 3.23	0.66	0.33	0.75	*a* = 3.21^c^	0.66	0.33	0.75		
	*c* = 5.18	Mg_2_	0.33	0.66	0.25	*c* = 5.25	0.33	0.66	0.25	*c* = 5.22	0.33	0.66	0.25	*c* = 5.21^c^	0.33	0.66	0.25		

a Experiment.^33^

b Experiment.^31^

c Experiment.^32^

The lattice constants of LiNaMg_2_H_6_, Li, Na and Mg compounds were calculated through variable-cell structural optimization by using the PW91, PBE and PBEsol functionals. According to previous experiments,^31–33^ it was assumed that the Li (Na) and Mg compounds adopt the cubic phase with the space group of *Im*3̄*m* and the hexagonal phase with the space group of *P*6_3_/*mmc*, respectively (see Fig. S3, SI). As shown in Table 1, the PBEsol-calculated lattice constant of LiNaMg_2_H_6_ is *a* = 7.60 Å, which is slightly smaller (larger) than the PBE(PW91)-calculated one of *a* = 7.62 Å (*a* = 7.58 Å). The PBEsol-calculated cell volume per formula unit is 109.74 Å^3^ per f.u., which is in excellent agreement with a previously calculated value of 109.66 Å^3^ per f.u.,^29^ but is slightly underestimated compared to a previous experimental result of 112.75 Å^3^ per f.u.,^28^ being attributed to the choice of a different crystalline structure. Meanwhile, the lattice constants optimized for Na, Li and Mg compounds are in good agreement with the previous experiments.^31–33^ In particular, the PBE-calculated lattice constants of *a* = 3.26, 4.25 and 3.49 Å (*c* = 5.25 Å) overestimate the experimental ones^31–33^ of *a* = 3.21, 4.22 and 3.47 Å (*c* = 5.21 Å), whereas the PW91-calculated ones of *a* = 3.16, 4.17 and 3.44 Å (*c* = 5.18 Å) slightly underestimate the experimental ones for Li, Na and Mg, respectively. Moreover, the PBEsol-calculated lattice constants of *a* = 3.23, 4.23 and 3.47 Å (*c* = 5.22 Å) are in excellent agreement with the experimental ones, giving a relative error of less than 0.5%. Hereafter, the PBEsol-optimized crystalline structures were adopted for the calculations of lattice dynamics and electronic structure properties, mechanical stability and H_2_ desorption energetics for the double-perovskite-type hydride LiNaMg_2_H_6_.

### 3.2 Electronic structure properties

**Fig. 2:**
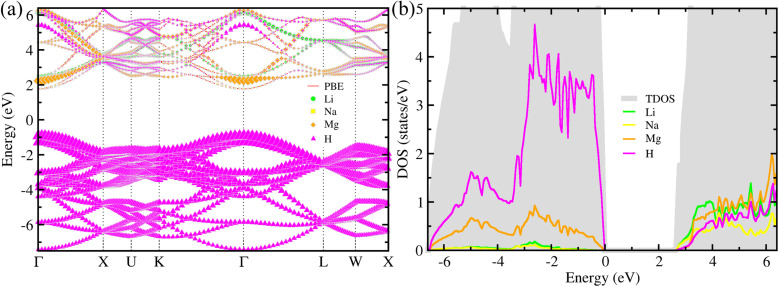
(a) Atom-resolved electronic band structure and (b) total and atom-projected partial density of states (DOS) calculated with the PBEsol functional for the double-perovskite-type hydride LiNaMg_2_H_6_ in the cubic phase. The green-, yellow-, brown- and magenta-colored symbols represent the Li, Na, Mg and H atom contributions to the energy band, respectively, and the size of them is proportional to the contribution amounts.

**Fig. 3:**
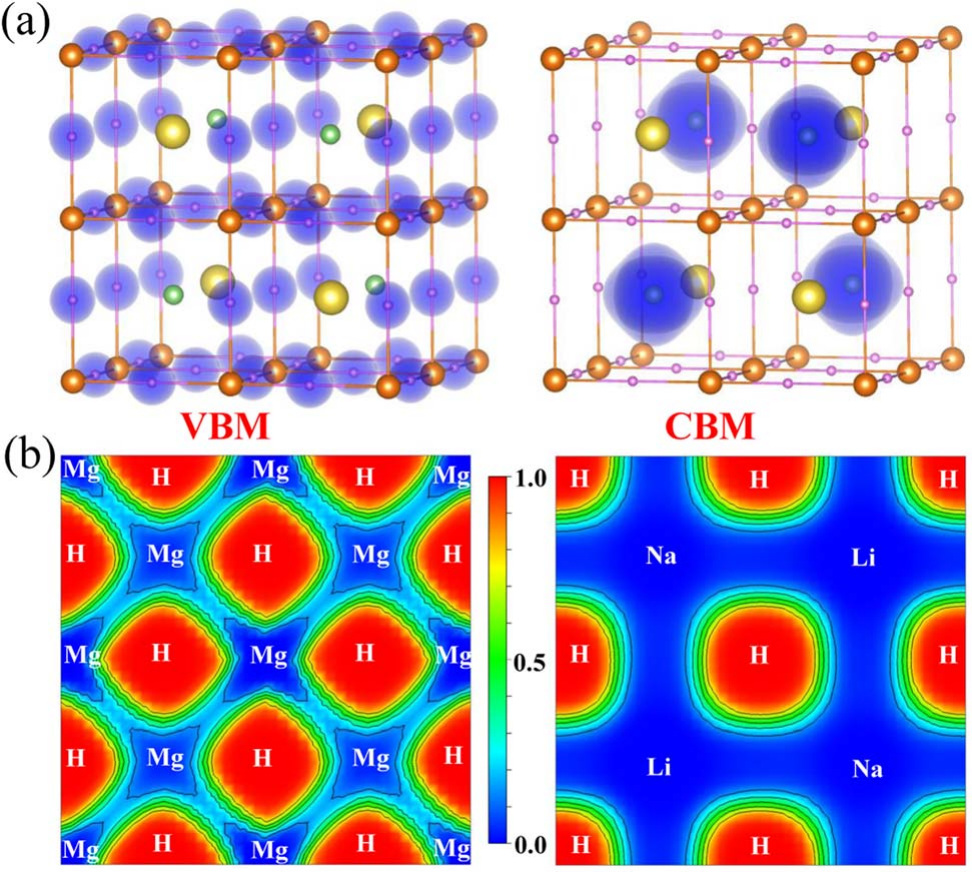
(a) Isosurface plot of electron charge density corresponding to the valence band maximum (VBM) and the conduction band minimum (CBM) at a value of 0.05 |*e*| Å^−3^. The green, yellow, brown and purple balls represent the Li, Na, Mg and H atoms, respectively. (b) Electron localization function (ELF) plots on two different surfaces centered by Mg and H atoms along the [001]-direction for LiNaMg_2_H_6_.

In the first step, we calculated the electronic structure properties, including the energy band structure, electron density of states (DOS) and charge density, for the double-perovskite-type hydride LiNaMg_2_H_6_ in the cubic phase with the *Fm*3̄*m* space group. As all constituent atoms are very light, spin–orbit coupling (SOC) interactions were not considered. Fig. 2(a) shows the PBEsol-calculated energy band structure along the high-symmetry line of *Γ*–*X*–*U*–*K*–*Γ*–*L*–*W*–*X* in the Brillouin zone for LiNaMg_2_H_6_. In the atom-resolved energy bands, the green-, yellow-, brown- and magenta-colored symbols represent the Li, Na, Mg and H atom contributions to the bands, respectively, and the size of the symbols is proportional to their contribution amounts. From Fig. 2(a), it was demonstrated that the cubic LiNaMg_2_H_6_ has a direct transition at the BZ center of the *Γ* point with a band gap of 2.8 eV, and an indirect transition from the W to the *Γ* point with a band gap of 3.3 eV, and is therefore regarded an insulator. On the other hand, in order to obtain a more accurate band gap, we calculated the energy band structure using the HSE06 hybrid functional, which leads to a larger band gap of 3.7 eV due to pushing up (down) the conduction (valence) bands (see Fig. S9, SI). Through the analysis of atom-projected partial DOS, it was revealed that the valence bands (VBs) are dominated by H atoms with a small contribution from Mg atoms, while the conduction bands (CBs) are characterized by a combination of Li, Na, Mg and H atomic contributions (see Fig. 2(b)). The Li and Na atoms make negligible contributions to the VBs, showing that the Li^+^ and Na^+^ cations simply function as spacers and electron donors in the cubic LiNaMg_2_H_6_. In addition, as shown in Fig. 3(a), the H-1s states make major contributions to the valence band maximum (VBM), while the Li-2s states play dominant roles in the conduction band minimum (CBM).

### 3.3 Chemical bonding properties

We then calculated chemical bonding properties such as electron localization function (ELF) and Born effective charge (BEC) for the double-perovskite-type hydride LiNaMg_2_H_6_ in the cubic phase with the *Fm*3̄*m* space group. The chemical bonding features can be identified by analyzing the electron localization function (ELF) and Born effective charge (BEC). In Fig. 3(b), we show the calculated ELF plots on two different surfaces centered by Mg and H atoms along the [001]-direction for LiNaMg_2_H_6_. The ELF values at the Li and Na sites were found to be nearly zero, whereas those at the H sites were close to one. Such a prominent difference in the ELF values implies that electrons are transferred from the metal atoms to the H atoms and few valence electrons are left between H and Li (Na) atoms, therefore indicating a high degree of ionic features for Li(Na)–H bonds. On the other hand, the ELF values of the Mg sites are slightly larger than zero, but still much smaller than those of the H sites, and a small amount of valence electrons is left between H and Mg atoms. It is obvious that, on the surface centered by Mg atoms, the ELF distributions around the Mg and H atoms are not isotropic, varying in different directions of the crystal. This implies that there must be a small amount of covalent character in the Mg–H bonds, but the ionic character is still significant. The ionic bonding nature leads to the insulating property of the LiNaMg_2_H_6_ hydride, consistent with the energy band structure and electron DOS calculations.

In an other attempt to judge the chemical bonding nature, we calculated the BEC using the Berry-phase approximation implemented in the VASP code. It was identified that the diagonal components of the BEC are same, satisfying the relation of *Z*_*xx*_ = *Z*_*yy*_ = *Z*_*zz*_, while the off-diagonal ones are zero at the Li, Na and Mg sites of LiNaMg_2_H_6_. This is natural for ionic compounds because of the spherical character in ionic bonds. Meanwhile, at the H sites, the diagonal components are slightly different, but almost equal and the off-diagonal ones are negligible. This is probably ascribed to some exchange due to sharing of electrons between H and Mg atoms. The LiNaMg_2_H_6_ hydride provides effective charges of +1.12, +1.34, +1.62, −0.95*e* for Na, Li, Mg and H atoms, respectively. The BEC analysis accordingly confirms that the Li (Na) and Mg atoms donate one and two electrons, respectively, whereas the H atom correspondingly gains one electron. The overlap populations between cations and anions are close to zero, as expected for ionic compounds. This BEC analysis is in good accordance with the ELF analysis.

### 3.4 Lattice dynamics properties

**Fig. 4:**
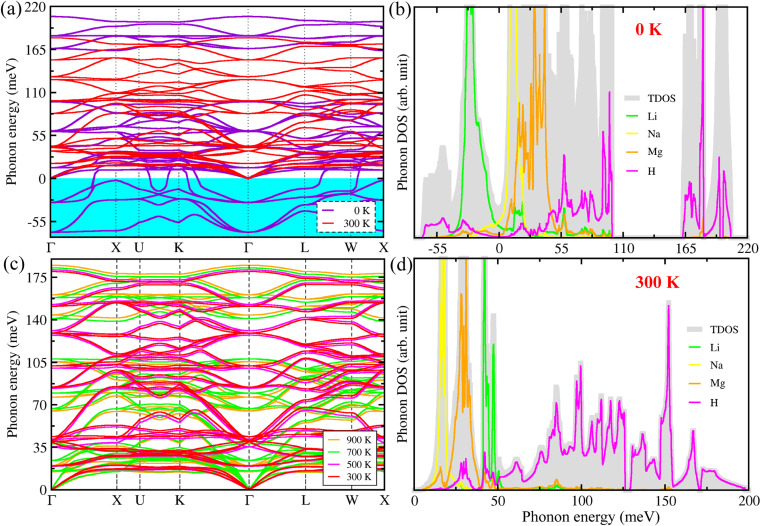
(a and c) Phonon dispersion curves and (b and d) atom-projected phonon density of states (DOS) calculated using the self-consistent phonon (SCP) theory at elevated temperatures for double-perovskite-type hydride LiNaMg_2_H_6_ in the cubic phase of space group *Fm*3̄*m*.

In the next step, we calculated the phonon dispersion curves and phonon density of states (DOS) at 0 K and finite temperatures by using the SCP theory, in order to estimate lattice dynamics properties for the cubic LiNaMg_2_H_6_ with the *Fm*3̄*m* space group. Fig. 4(a) shows the harmonic phonon dispersion curves (purple-colored lines) computed at 0 K along the high-symmetry line of Γ–X–U–K–Γ–L–W–X in the phonon Brillouin zone (BZ). We found that the phonon dispersions have relatively deep negative phonon eigenvalues reaching towards −62 meV (known as the phonon soft mode), crossing the whole range of the phonon BZ for the cubic LiNaMg_2_H_6_. The presence of the phonon soft modes implies that the double-perovskite-type hydride LiNaMg_2_H_6_ has strong lattice anharmonicity and is dynamically unstable in the cubic phase at 0 K like the halide and oxide perovskites. For instance, Zhao *et al.*^54^ showed that the lead-free halide double perovskites are dynamically unstable at 0 K, but can be subsequently stabilized by anharmonic phonon–phonon interactions above room temperature. Moreover, Klarbring *et al.*^55^ theoretically proved that the experimentally observed cubic phase of the halide double perovskite Cs_2_AgBiBr_6_ becomes dynamically stable due to the collapse of the soft phonon modes at finite temperatures. In the case of the oxide perovskite SrTiO_3_,^46^ it was also revealed that the unstable phonon modes occur at the Γ and R points of the harmonic phonon dispersion (*T* = 0 K), but in the anharmonic dispersions, these soft modes are renormalized to become real, confirming the dynamical stability of the cubic phase at finite temperatures. From the atom-projected phonon DOS at 0 K (Fig. 4(b)), it was found that these soft phonon modes are mainly ascribed to the H- and Li-atomic vibrations for the LiNaMg_2_H_6_. Based on detailed analysis of the phonon eigenvectors, we find that the unstable phonon modes at the Γ (X) point are responsible for symmetry-breaking instabilities causing the ferroelectric (anti-ferroelectric) displacement of Li atoms (MgH_6_ octahedra tilting). That is, a lower-symmetry orthorhombic LiNaMg_2_H_6_ with *Pnma* space group can be stabilized at low temperatures (see Fig. S7, SI) like in NaMgH_3_,^6^ but it is not suitable for H_2_ storage application at high temperatures. The calculated phonon dispersion curves and phonon DOS were plotted in Fig. S4–S6, SI, for cubic Li and Na and hexagonal Mg without any unstable phonon modes, implying that they are dynamically stable at 0 K.

For the purpose of estimating the dynamical stability of the cubic phase at finite temperatures, we calculated the phonon dispersion curves and DOS at elevated temperatures from 300 to 900 K with a step size of 200 K by considering the anharmonic phonon–phonon interactions based on the SCP theory. As can be seen in Fig. 4(a and c), the eigenvalues corresponding to the soft phonon modes shown in the harmonic dispersion curves were clearly renormalized to be real within the whole range of the phonon BZ at finite temperatures over 300 K. This indicates that the cubic phase of LiNaMg_2_H_6_ is dynamically stable at elevated temperatures, like in the case of the halide and oxide perovskite compounds. In particular, the negative phonon energies of −62 and −27 meV were renormalized to 32 and 45 meV at the Γ point and *T* = 300 K for the cubic LiNaMg_2_H_6_. In the temperature-dependent phonon dispersions, noticeable changes were also observed for the high-lying optical phonon modes. Compared to the harmonic phonon dispersions, the high-energy optical modes pushed downward and, therefore, the phonon energy gap between 100 and 163 meV disappeared for the anharmonic phonon dispersions at temperatures over 300 K. As shown in Fig. 4(d), the Li, Na and Mg atoms play dominant roles in the acoustic mode coupled with the low-lying optical phonon modes below 50 meV, whereas the H atoms make significant contributions to the mid- and high-lying optical phonon modes above 50 meV at 300 K for the cubic LiNaMg_2_H_6_. On the other hand, with the increment of temperature, the phonon dispersion curves broaden towards higher energy because of stronger atomic vibrations, coinciding with the general knowledge of lattice vibrations (see Fig. 4(c)). Additionally, we calculated heat capacity, internal energy, entropy and Helmholtz free energy of the cubic LiNaMg_2_H_6_ as functions of temperature (see Fig. S8, SI), demonstrating its excellent thermodynamic stability at high temperatures because the free energy decreases as the temperature increases.

### 3.5 Elastic properties and H_2_ desorption energetics

In the final stage, we estimated the elastic properties and mechanical stability by calculating elastic constants such as the stiffness constant (*C*_*ij*_), bulk modulus (*B*), shear modulus (*G*) and Young’s modulus (*E*) using DFPT calculations. The elastic constants play an important role in estimating the resistance of materials against elastic deformation. In Table 2, we show the three calculated independent elastic stiffness constants, namely, *C*_11_, *C*_12_ and *C*_44_ for the single-crystalline LiNaMg_2_H_6_. As can be seen, the elastic stiffness constants satisfy the Born’s stability criteria for the cubic phase, confirming the mechanical stability for LiNaMg_2_H_6_. The shear elastic constant *C*_44_ is more than 50% smaller than the unidirectional component *C*_11_, indicating that the resistance to shear deformation is much weaker than that to unidirectional deformation. The strength of the polycrystalline solid can be estimated by its elastic bulk (*B*), shear (*G*) and Young’s (*E*) moduli, which define the response to uniform, shear and uniaxial stress, respectively. *E* has the largest value among the three moduli, implying that the resistance to uniaxial deformation is stronger than the ones to uniform and shear deformations. Furthermore, it was found that the cubic LiNaMg_2_H_6_ is a brittle material according to the Pugh’s criteria^56^ because the Poisson’s ratio *ν* and the Pugh’s ratio *B*/*G* are smaller than the threshold values of 0.26 and 1.75, respectively (see Table 2). Our calculated *B*, *G* and *E* values of 39.76, 28.04 and 68.10 GPa for LiNaMg_2_H_6_ are in reasonable agreement with other theoretical results^22^ of 34.04, 29.63 and 68.89 GPa for the analogous double-perovskite-type hydride KNaMg_2_H_6_, where Li atoms are substituted by K atoms. In comparison with LiMgH_3_ and NaMgH_3_, the computed *B* value of 39.76 GPa for LiNaMg_2_H_6_ is slightly larger (smaller) than the value of 38.4 (39.8) GPa for LiMgH_3_ (NaMgH_3_).^53^ In addition, the LiNaMg_2_H_6_ hydride is completely isotropic in its elastic behavior because the values of *A*^*U*^, *A*_C_ and *A*_S_ are zero, and the value of *A*_1_ is nearly unity.

**Table 2:** Gravimetric and volumetric H_2_ storage density (*ρ*_g_ and *ρ*_v_), elastic stiffness constant (*C*_*ij*_), bulk modulus (*B*), shear modulus (*G*), Young’s modulus (*E*), Pugh’s ratio (*B*/*G*), Poisson’s ratio (*ν*), band gap (*E*_g_), elastic anisotropic factors of *A*^*U*^, *A*_C_, *A*_S_ and *A*_1_, zero-point energy (*E*_*z*_), H_2_ decomposition enthalpy (Δ*H*) and decomposition temperatures (*T*_des_ and *T*^q^_des_ without and with quantum effect) for the cubic LiNaMg_2_H_6_

Functional	Properties	LiNaMg_2_H_6_	Li	Na	Mg	H_2_
	*ρ*_g_ (wt%)	7.09				
	*ρ*_v_ (g L^−1^)	91.12				
PW91	*H* (eV)	−28.42	−3.77	−2.61	−2.58	−6.81
	Δ*H* (eV)	2.22				
	*T*_des_ (K)	545.81				
PBE	*H* (eV)	−28.21	−3.81	−2.62	−2.48	−6.77
	Δ*H* (eV)	2.20				
	*T*_des_ (K)	541.66				
PBEsol	*H* (eV)	−28.22	−3.92	−2.80	−2.98	−6.51
	Δ*H* (eV)	2.34				
	*T*_des_ (K)	575.67				
	*E*_*z*_ (eV)	1.00	0.03	0.02	0.06	0.27
	*T* ^q^_des_ (K)	548.54				
	*E* ^PBEsol^_g_ (eV)	2.8				
	*E* ^HSE^_g_ (eV)	3.7				
	*C*_11_ (GPa)	77.60				
	*C*_12_ (GPa)	20.84				
	*C*_44_ (GPa)	27.81				
	*B* (GPa)	39.76				
	*G* (GPa)	28.04				
	*E* (GPa)	68.10				
	*B*/*G*	1.42				
	*ν*	0.21				
	*A* ^*U*^	0.00				
	*A*_C_	0.00				
	*A*_S_	0.00				
	*A*_1_	0.98				

Finally, we estimated the H_2_ storage capacities and H_2_ desorption energetics for the double-perovskite-type hydride LiNaMg_2_H_6_. The H_2_ gravimetric and volumetric storage capacities were computed by using the formulas of *ρ*_g_ = 6*M*_H_/(*M*_Li_ + *M*_Na_ + 2*M*_Mg_ + 6*M*_H_) × 100% and *ρ*_v_ = 6*M*_H_/(*N*_A_·*V*_opt_), where *M*_Li_, *M*_Na_, *M*_Mg_ and *M*_H_ are the molar masses of Li, Na, Mg and H atoms, respectively, whereas *N*_A_ and *V*_opt_ are the Avogadro number and PBEsol-optimized unit cell volume. As listed in Table 2, LiNaMg_2_H_6_ has high *ρ*_g_ and *ρ*_v_ values of 7.09 wt% and 91.12 g L^−1^, respectively, which are much larger than the targeted values of 5.5 wt% and 40 g L^−1^ provided by the U.S. DOE.

Moreover, LiNaMg_2_H_6_ has higher H_2_ storage capacities compared with other perovskite-type hydrides of Mg_2_FeH_6_ (5.47 wt%)^11^ and MMgH_3_ (M = Na, K, Rb) (<6.00 wt%).^16^ Finally, we calculated the H_2_ desorption temperature *T*^q^_des_ and *T*_des_ with and without considering the quantum effect by using eqn (5). Without consideration of the quantum effect, the PW91-, PBE- and PBEsol-calculated *T*_des_ values are 545.81, 541.66 and 575.67 K, respectively, for the LiNaMg_2_H_6_ hydride. In a previous experiment,^30^ Wang *et al.* observed that the Li_0.5_Na_0.5_MgH_3_ hydride has the lowest H_2_ desorption temperature of ∼580 K among the Li_*x*_Na_1−*x*_MgH_3_ hydrides, which is in good agreement with the PBEsol-calculated one of 575.67 K. By using the harmonic phonon energies and DOS, we estimated the zero-point energy *E*_*z*_, and considered the quantum effect for the calculation of the H_2_ desorption temperature. It was found that by considering the quantum effect, the *T*^q^_des_ value decreased slightly to 548.54 K for LiNaMg_2_H_6_. In order to consider the anharmonic effect on the *T*^q^_des_ calculation, we also estimated *E*_*z*_ using the SCP energies and DOS, and then recalculated *T*^q^_des_, demonstrating that the anharmonic effect slightly decreases *T*^q^_des_ to 538.19 K. In addition, we estimated an uncertainty of the calculated *T*^q^_des_ by using the uncertainty of the H_2_ desorption enthalpy Δ*H*_H_2__ in eqn (5), leading to *T*^q^_des_ = 538.19 ± 24.59 K.

For the purpose of discussing kinetic properties for the H_2_ absorption–desorption processes, we calculated the activation energies for H_2_ migration in LiNaMg_2_H_6_ and NaMgH_3_ by employing the climbing image nudged elastic band (NEB) method. As shown in Fig. S10, SI, LiNaMg_2_H_6_ has an activation energy of 0.31 eV, lower than the value of 0.46 eV for NaMgH_3_. This result directly implies that substituting Na atoms with Li atoms leads to better kinetic properties. To sum up, the double-perovskite-type hydride LiNaMg_2_H_6_ can store 7.09 wt% and 91.12 g L^−1^ hydrogen, exhibits mechanical and dynamical stability, and has a suitable H_2_ desorption temperature of about 540 K, satisfying the U.S. DOE requirement.

## 4 Conclusions

In conclusion, by using density functional theory calculations, we have provided theoretical insights into the material properties, such as structural, electronic and lattice dynamics properties, and mechanical and dynamical stabilities, of the double-perovskite-type hydride LiNaMg_2_H_6_ in the cubic phase of the *Fm*3̄*m* space group for H_2_ storage applications. Based on the analysis of the Goldschmidt tolerance factor *t*_G_ and octahedral factor *t*_o_, it was suggested that the LiNaMg_2_H_6_ hydride can stabilize in a perovskite-type crystalline structure. It was found that the PBEsol-calculated lattice constants and atomic positions are in good accordance with the available experimental data, while the PBE(PW91) functional slightly overestimates (underestimates) the structural properties for Li, Na, Mg and LiNaMg_2_H_6_. From the electronic structure calculations, it was demonstrated that cubic LiNaMg_2_H_6_ has a direct (indirect) band gap of 2.8 (3.3) eV at the Γ point (with the VBM at the W point and the CBM at the Γ point). The atom-projected electron partial DOS indicated that the H atoms make major contributions to the valence bands, while a combination of Li, Na, Mg and H atomic contributions dominates in the conduction bands. The harmonic phonon dispersions and phonon DOS calculations demonstrated that the cubic phase is dynamically unstable at 0 K, with the negative phonon energies crossing the phonon BZ. However, the negative phonon energies were renormalized to be real by considering the anharmonic phonon–phonon interactions within the SCP approach, indicating that LiNaMg_2_H_6_ can be dynamically stabilized in the cubic phase at elevated temperatures. From the DFPT calculations on the elastic constants, it was revealed that the cubic phase of LiNaMg_2_H_6_ is mechanically stable, satisfying the Born’s stability criteria. Finally, we investigated the H_2_ storage capacities and desorption temperature by considering the quantum effect, finding that *ρ*_g_ (*ρ*_v_) is 7.09 wt% (91.12 g L^−1^), and *T*^q^_des_ is 548.54 K for LiNaMg_2_H_6_. It should be noted that our calculations on the structural and elastic properties and the H_2_ desorption temperature explain well the previous experimental results, uncovering the atomistic insights into the material properties for LiNaMg_2_H_6_. Based on such calculations, it was concluded that the double-perovskite-type hydride LiNaMg_2_H_6_ is a potential candidate for the onboard H_2_ storage application with high gravimetric and volumetric capacities, high mechanical and dynamical stabilities, and a suitable dehydrogenation temperature.

## Author contributions

Son-Il Jo, Hyong-Ju Kim and Un-Gi Jong developed the original project, performed the DFT calculations and drafted the first manuscript. Tal-Hwan Gye assisted with the post-processing of the computational data. Un-Gi Jong and Son-Il Jo supervised the work. All authors reviewed the manuscript.

## Conflicts of interest

There are no conflicts to declare.

## Supplementary Material

RA-015-D5RA05174F-s001

## Data Availability

Data are available on the reasonable request to the corresponding author. Supplementary information: total energy convergence tests according to the kinetic energy cutoff and *k*-point mesh, crystalline structures, phonon dispersion curves and phonon DOS for the cubic Li and Na, and the hexagonal Mg compounds, comparison of atomic forces estimated from the DFT and CSLD calculations, HSE06-calculated band structure, and Helmholtz free energy. See DOI: https://doi.org/10.1039/d5ra05174f.
