# Supplementary material for: Atomic insights into the material properties of double-perovskite-type hydride LiNaMg2H6 for H2 storage applications
Source: RSC Adv. 2025 Oct 16;15(46):38906–15. doi: 10.1039/d5ra05174f (PMC12529092; doi:10.1039/d5ra05174f)
Supplement: RA-015-D5RA05174F-s001 [file RA-015-D5RA05174F-s001.pdf]

Supplementary Information (SI) for RSC Advances.
This journal is © The Royal Society of Chemistry 2025

Electronic Supplementary Information (ESI) – Atomic insights into materials properties of double perovskite-type hydride $LiNaMg_2H_6$ for H_2 storage applications

Son-Il Jo,[a*] Hyong-Ju Kim,[a] Chol-Ho Pang,[a] Un-Gi Jong,[b†] Tal-Hwan Kye,[a]

[a] *Faculty of Materials Science and Technology, Kim Chaek University of Technology, Pyongyang, PO Box 76, Democratic People's Republic of Korea.*
[b] *Faculty of Materials Science, Kim Il Sung University, Pyongyang, PO Box 76, Democratic People's Republic of Korea.*

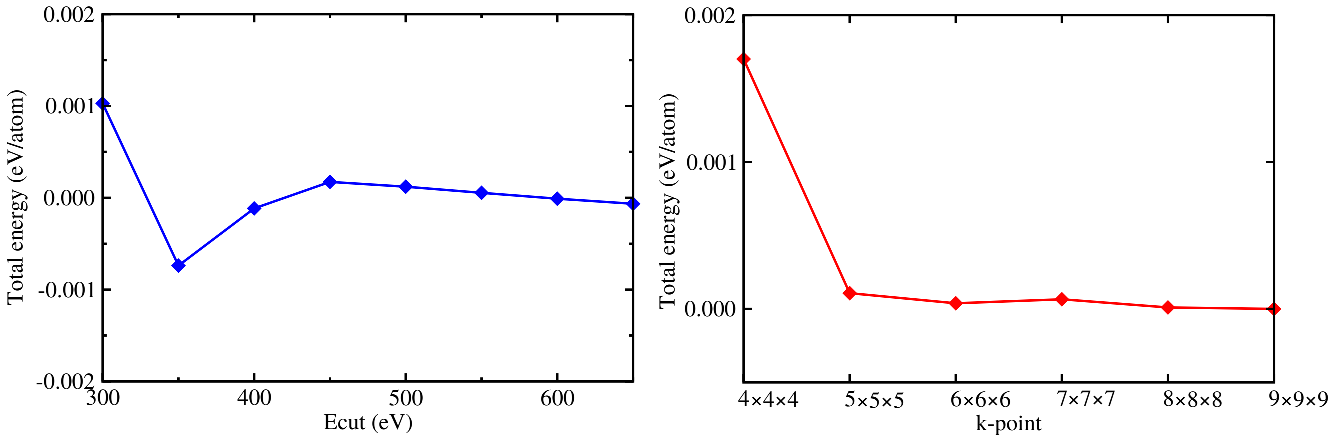

Figure S1 Convergence tests of total energy per atom for a primitive unit cell containing 10 atoms according to the sizes of the energy cutoff and *k*-point mesh for the double perovskite-type perovskite $LiNaMg_2H_6$.

*Son-Il Jo, Email: jsi85922@star-co.net.kp
†Un-Gi Jong, Email: ug.jong@ryongnamsan.edu.kp

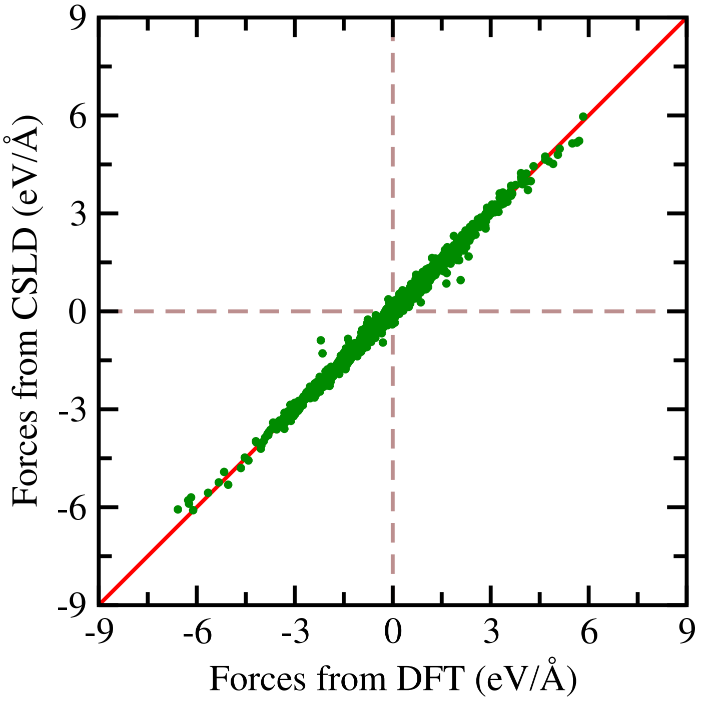

Figure S2 Comparison of atomic forces estimated from the DFT versus CSLD calculations for the double perovskite-type perovskite $LiNaMg_2H_6$.

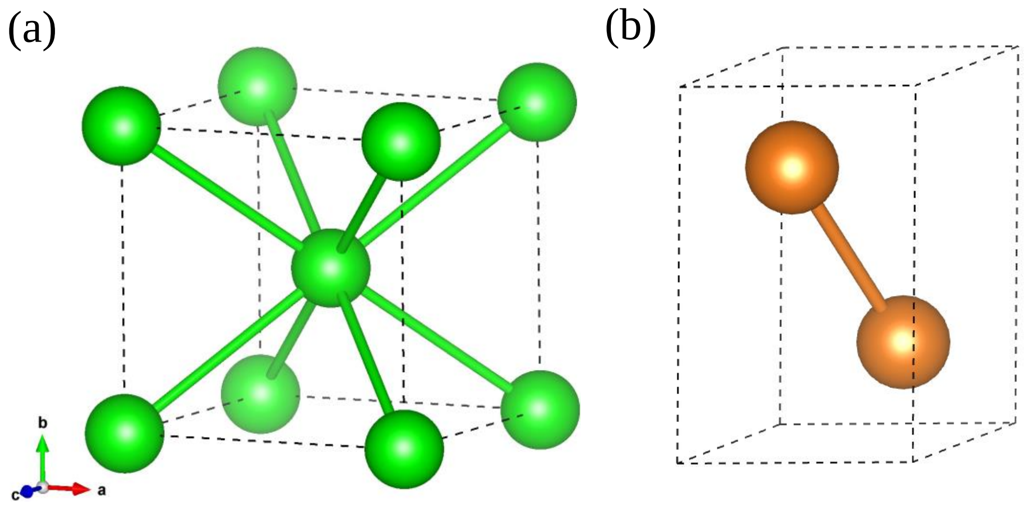

Figure S3 Crystalline structure for (a) the cubic Li (Na) with the $Im\bar{3}m$ space group and (b) the hexagonal Mg with the $P63/mmc$ space group.

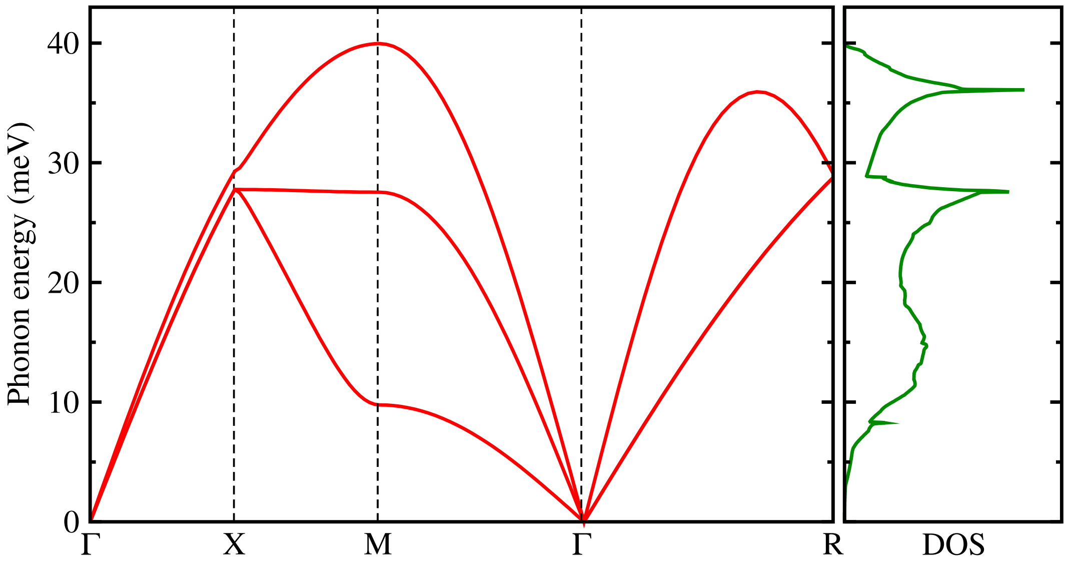

Figure S4 Phonon dispersion curves and phonon density of states (DOS) for the cubic Li with the $Im\bar{3}m$ space group.

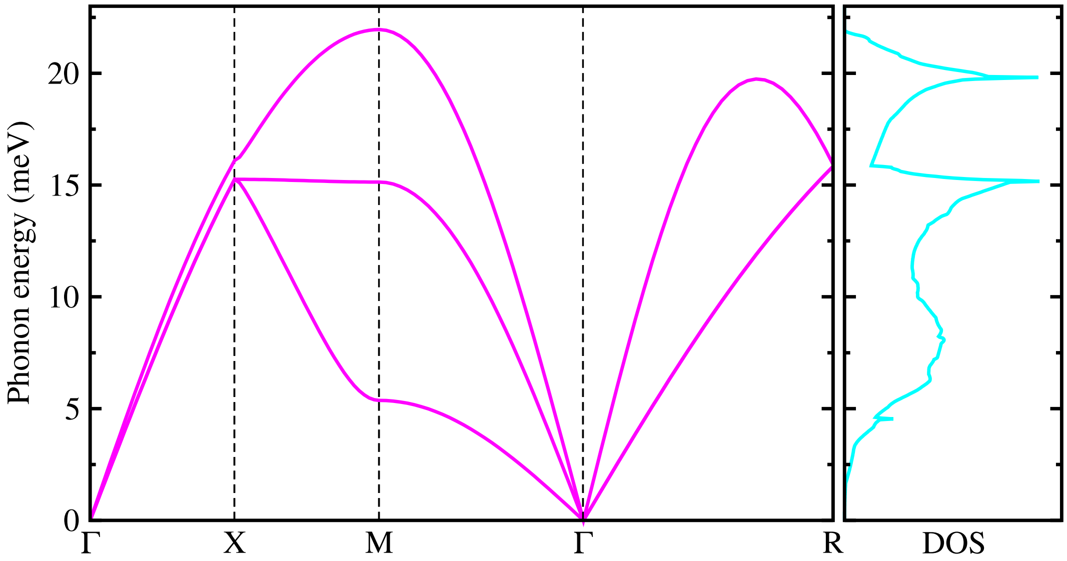

Figure S5 Phonon dispersion curves and phonon density of states (DOS) for the cubic Na with the $Im\bar{3}m$ space group.

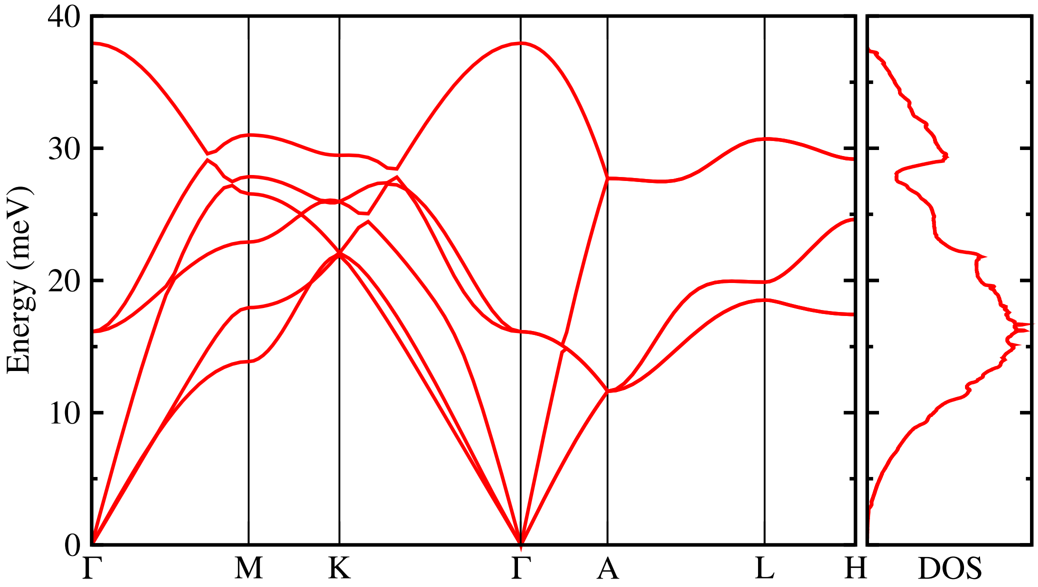

Figure S6 Phonon dispersion curves and phonon density of states (DOS) for the hexagonal Mg with the *P*63/*mmc* space group.

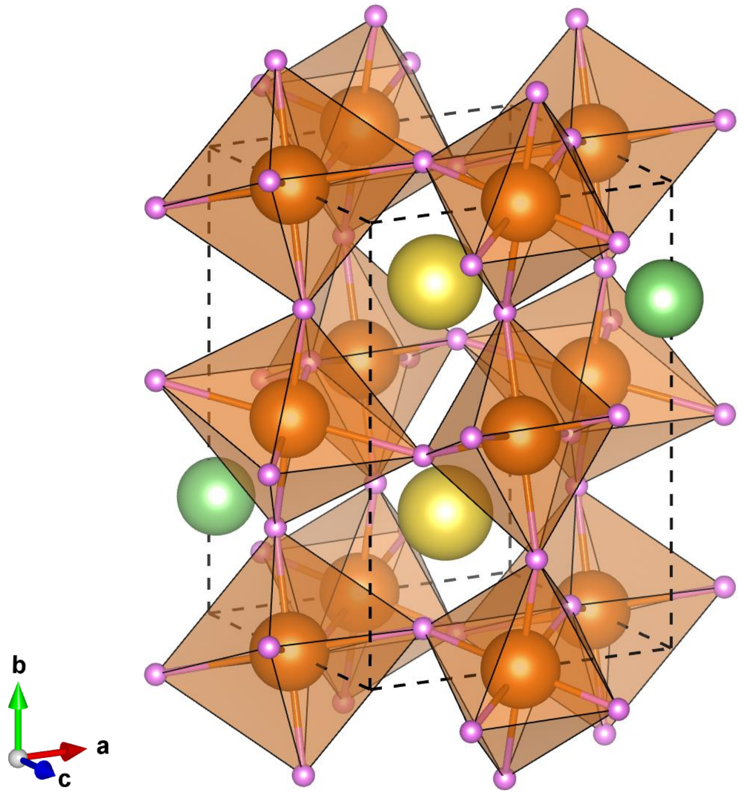

Figure S7 Polyhedral view of crystalline structure optimized using PBEsol functional for double perovskite-type hydride $LiNaMg_2H_6$ in orthorhombic phase with a space group of *Pnma*. The green-, yellow-, brown- and purple-colored balls represent the Li, Na, Mg and H atoms, respectively.

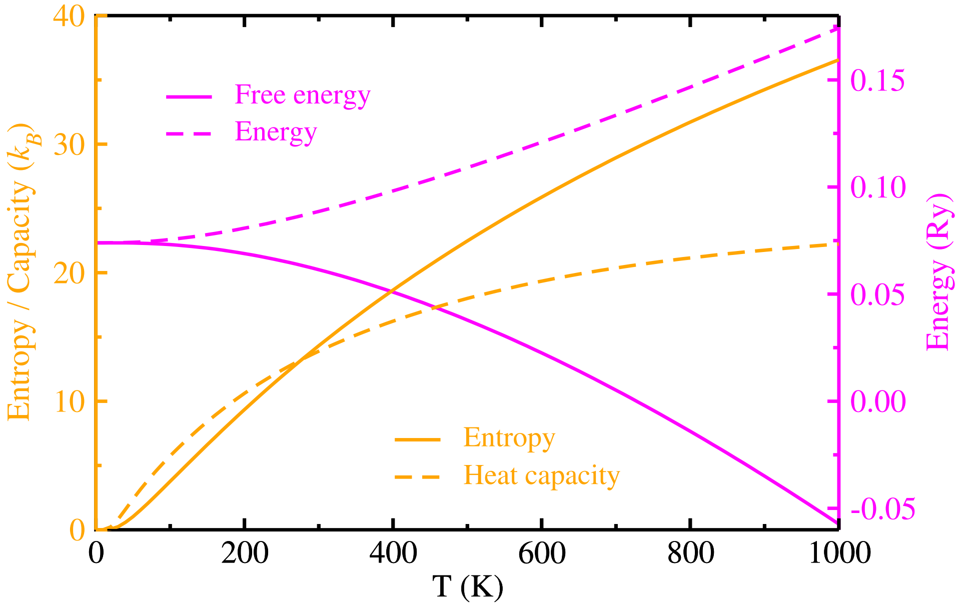

Figure S8 Heat capacity, internal energy, entropy and Helmholtz free energy of the cubic $LiNaMg_2H_6$ as functions of temperature.

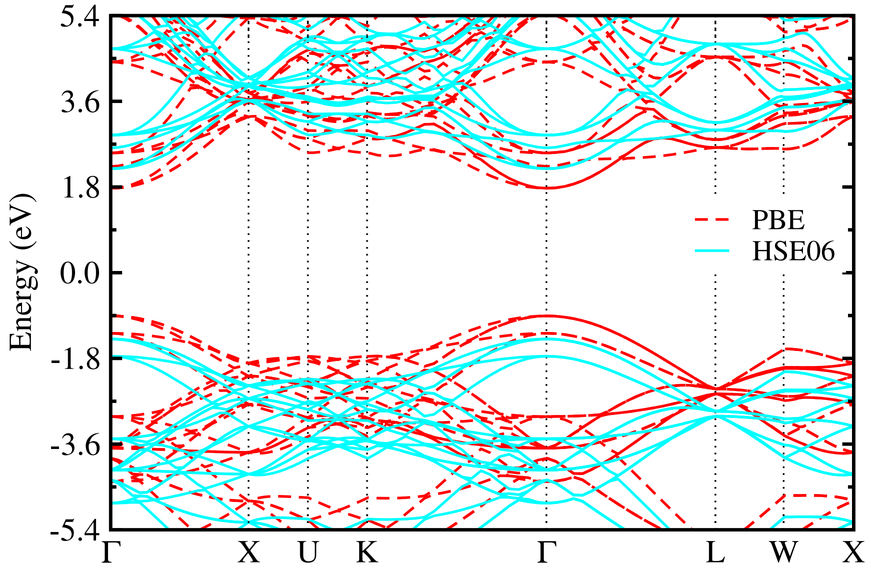

Figure S9 Comparison of energy band structures calculated with PBE and HSE06 functionals for the cubic $LiNaMg_2H_6$.

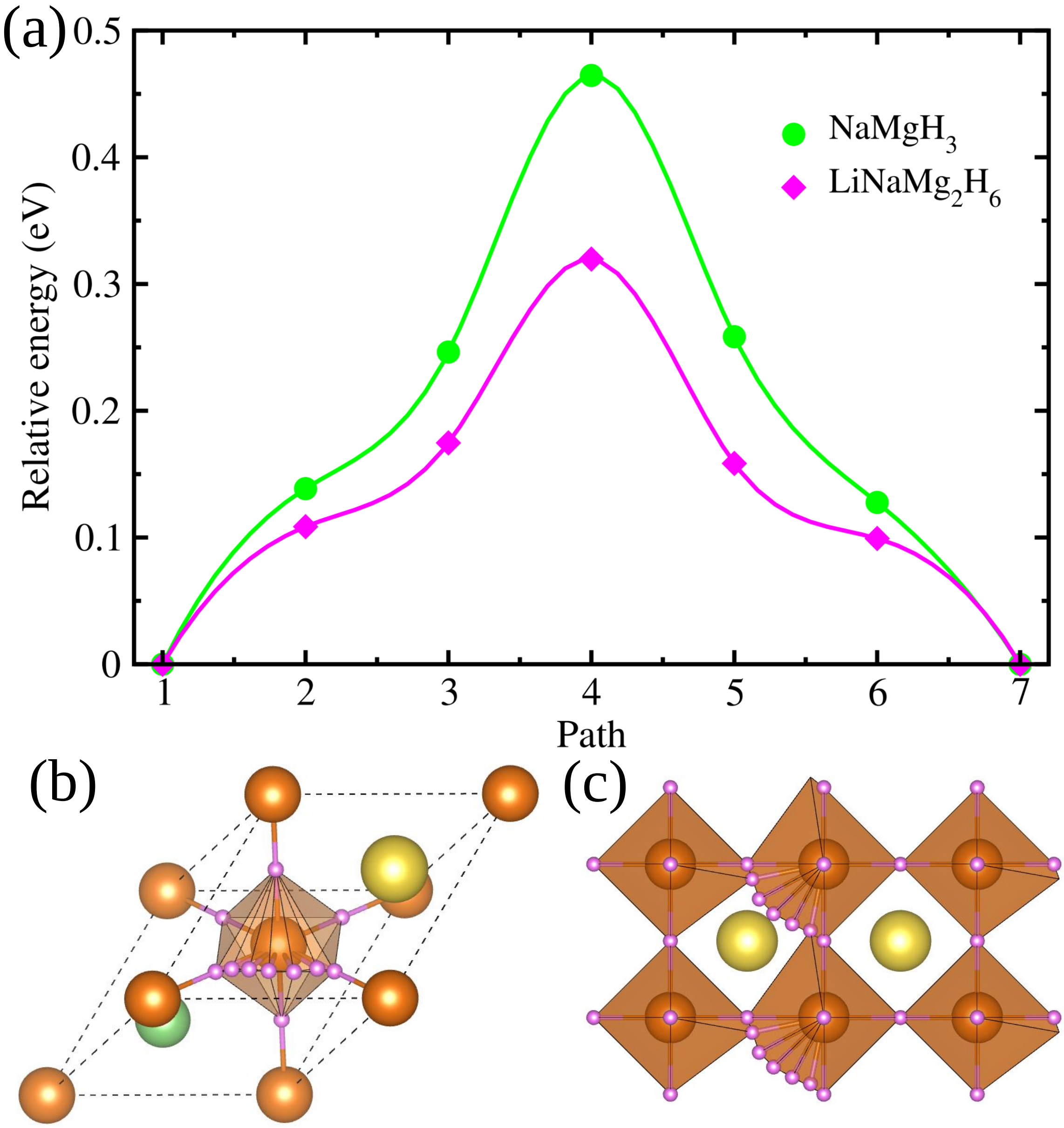

Figure S10 (a) Activation energy profile corresponding for H_2 migration in $LiNaMg_2H_6$ and $NaMgH_3$, (b) and (c) migration paths for $LiNaMg_2H_6$ and $NaMgH_3$. The green-, yellow-, brown- and purple-colored balls represent the Li, Na, Mg and H atoms, respectively.
